# Substrate-specific effects of pirinixic acid derivatives on ABCB1-mediated drug transport

**DOI:** 10.18632/oncotarget.7345

**Published:** 2016-02-12

**Authors:** Martin Michaelis, Florian Rothweiler, Mario Wurglics, Natália Aniceto, Michaela Dittrich, Heiko Zettl, Michael Wiese, Mark Wass, Taravat Ghafourian, Manfred Schubert-Zsilavecz, Jindrich Cinatl

**Affiliations:** ^1^ Institut für Medizinische Virologie, Klinikum der Goethe-Universität, Frankfurt am Main 60596, Germany; ^2^ Centre for Molecular Processing and School of Biosciences, University of Kent, Canterbury CT2 7NJ, UK; ^3^ Institute for Pharmaceutical Chemistry, Goethe-University, Frankfurt am Main 60438, Germany; ^4^ Medway School of Pharmacy, Universities of Kent and Greenwich in Medway, Chatham, Kent ME4 4TB, UK; ^5^ Pharmaceutical Institute, University of Bonn, Bonn 53121, Germany; ^6^ School of Life Sciences, University of Sussex, Brighton BN1 9QG, UK; ^7^ Current address: Centre for Molecular Processing and School of Biosciences, University of Kent, Canterbury CT2 7NJ, UK

**Keywords:** pirinixic acid derivative, ABCB1, cancer, drug resistance, pirinixic acid

## Abstract

Pirinixic acid derivatives, a new class of drug candidates for a range of diseases, interfere with targets including PPARα, PPARγ, 5-lipoxygenase (5-LO), and microsomal prostaglandin and E2 synthase-1 (mPGES1). Since 5-LO, mPGES1, PPARα, and PPARγ represent potential anti-cancer drug targets, we here investigated the effects of 39 pirinixic acid derivatives on prostate cancer (PC-3) and neuroblastoma (UKF-NB-3) cell viability and, subsequently, the effects of selected compounds on drug-resistant neuroblastoma cells. Few compounds affected cancer cell viability in low micromolar concentrations but there was no correlation between the anti-cancer effects and the effects on 5-LO, mPGES1, PPARα, or PPARγ. Most strikingly, pirinixic acid derivatives interfered with drug transport by the ATP-binding cassette (ABC) transporter ABCB1 in a drug-specific fashion. LP117, the compound that exerted the strongest effect on ABCB1, interfered in the investigated concentrations of up to 2μM with the ABCB1-mediated transport of vincristine, vinorelbine, actinomycin D, paclitaxel, and calcein-AM but not of doxorubicin, rhodamine 123, or JC-1. *In silico* docking studies identified differences in the interaction profiles of the investigated ABCB1 substrates with the known ABCB1 binding sites that may explain the substrate-specific effects of LP117. Thus, pirinixic acid derivatives may offer potential as drug-specific modulators of ABCB1-mediated drug transport.

## INTRODUCTION

Pirinixic acid (WY-14,643) was discovered as a peroxisome proliferator-activated receptor (PPAR)α agonist in 1974 [1] and is related to the fibrates that are in clinical use for the treatment of hypercholesterolemia and hypertriglyceridemia [2]. In the meantime, a substantial number of pirinixic acid derivatives were synthesized that interfere with targets including PPARα, PPARγ, 5-lipoxygenase (5-LO), cyclooxygenase (COX), microsomal prostaglandin E2 synthase-1 (mPGES1), and γ-secretase at varying potencies [3–8]. This makes pirinixic acid derivatives drug candidates for pathological states including dyslipidemia, diabetes, metabolic syndrome, hypertension, cardiovascular disease, Alzheimer's disease, and inflammation-related diseases [2,9–13].

5-LO, mPGES1, PPARα, and PPARγ are also potential drug targets for anti-cancer therapies [14–17]. However, pirinixic acid derivatives had not been tested for anti-cancer activity, yet. Thus, we here investigated 39 pirinixic acid derivatives for their effects on the viability of a prostate cancer (PC-3) and a neuroblastoma (UKF-NB-3) cell line and, subsequently, the effects of selected compounds on drug-resistant neuroblastoma cells. Few compounds affected cancer cell viability in low micromolar concentrations but there was no correlation between the anti-cancer effects and the effects on 5-LO, mPGES1, PPARα, or PPARγ. Most strikingly, pirinixic acid derivatives interfered with drug transport by the ATP-binding cassette (ABC) transporter ABCB1 (also known as P-glycoprotein or MDR1) in a drug-specific fashion, i.e. they interfered with the ABCB1-mediated transport of only a subset of the investigated ABCB1 substrates.

## RESULTS

### Effects of pirinixic acid derivatives on cancer cell viability

**Figure 1:**
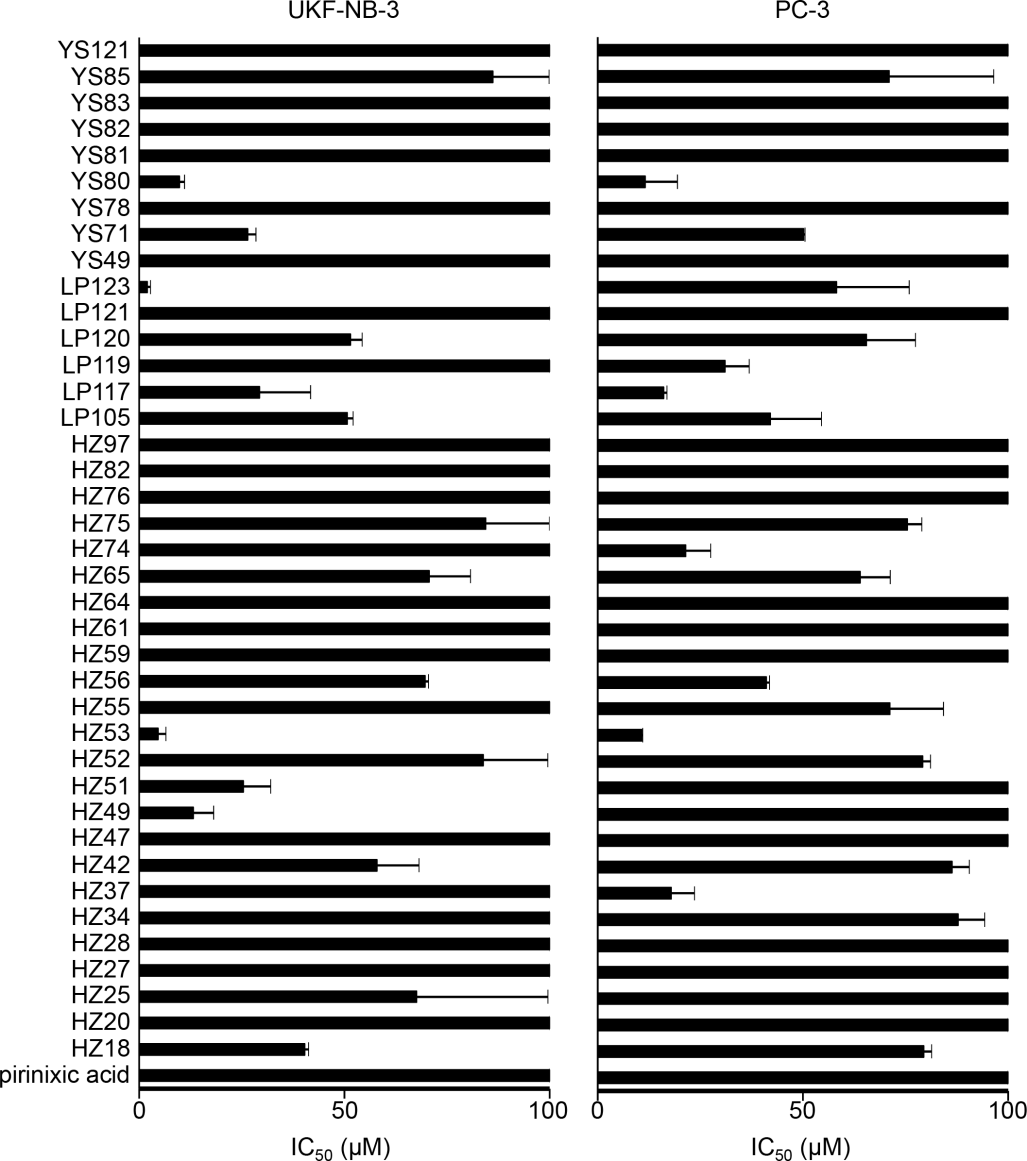
Effects of pirinixic and its derivatives on UKF-NB-3 neuroblastoma and PC-3 prostate cancer cell viability Concentrations that reduce cell viability by 50% (IC_50_) were determined after 120h of incubation by MTT assay. The corresponding numerical values are presented in Suppl. Table 1.

The effects of pirinixic acid and its 39 derivatives were determined on UKF-NB-3 neuroblastoma and PC-3 prostate carcinoma cell viability (Figure 1, Suppl. Table 1). To compare the effects of the compounds in the two cell lines they were classified according to their activities into compounds that did not reduce cell viability by 50% (IC_50_) in the tested concentration range up to 100μM, compounds that displayed IC_50_s between 10μM and 100μM, and compounds that displayed IC_50_s below 10μM. Although there were substantial similarities between the effects of most compounds on the viability of both cell lines, there were also some substantial differences (Figure 1, Suppl. Table 1).

18 compounds including pirinixic acid showed IC_50_ values >100μM in both cell lines. 11 compounds displayed IC_50_ values between 10μM and 100μM in UKF-NB-3 and PC-3 cells. Three compounds (HZ25, HZ49, HZ51) showed IC_50_ values between 10μM and 100μM in UKF-NB-3 cells but IC_50_s >100μM in PC-3 cells. Five compounds (HZ34, HZ37, HZ55, HZ74, LP119) displayed IC_50_ values <100μM and >10μM in PC-3 cells but IC_50_ values >100μM in UKF-NB-3. Three compounds (HZ53, LP123, YS80) had IC_50_ values <10μM in UKF-NB-3 cells and IC_50_ values >10μM and <100μM in PC-3 cells. Notably, there was no significant difference between the IC_50_ value of YS80 in UKF-NB-3 cells (9.87 ± 1.19μM) and in PC-3 cells (11.61 ± 7.83μM) although they were classified into different categories (Figure 1, Suppl. Table 1).

Compounds that were >2 times more effective in UKF-NB-3 cells than in PC-3 cells (fold change IC_50_ PC-3/IC_50_ UKF-NB-3 >2) included HZ49 (fold change >7.5), HZ51 (fold change 3.9), HZ53 (fold change 2.3), and LP123 (fold change 28.5). Compounds that were >2 times more effective in PC-3 than in UKF-NB-3 cells (fold change IC_50_ UKF-NB-3/IC_50_ PC-3 >2) included HZ37 (fold change >5.6), HZ74 (fold change >4.7), and LP119 (fold change >3.2). The lowest IC_50_ value was determined for LP123 in UKF-NB-3 cells (2.04 ± 0.69μM) (Figure 1, Suppl. Table 1).

### Effects of selected pirinixic acid derivatives on the viability of drug-resistant neuroblastoma cells

Next we compared the effects of pirinixic acid, HZ51, LP117, LP123, YS71, and YS80 on the viability of the cell line UKF-NB-3 and its sub-lines with acquired resistance to cisplatin (UKF-NB-3^r^CDDP^1000^), doxorubicin (UKF-NB-3^r^DOX^20^), and vincristine (UKF-NB-3^r^VCR^10^). In addition, we determined the effects of these compounds on the viability of Be(2)-C cells. Be(2)-C is a clonal sub-line of the neuroblastoma cell line SK-N-BE(2) that was isolated from a neuroblastoma patient after repeated courses of chemotherapy and radiotherapy [18] and that displays multi-drug resistance [19,20].

**Figure 2:**
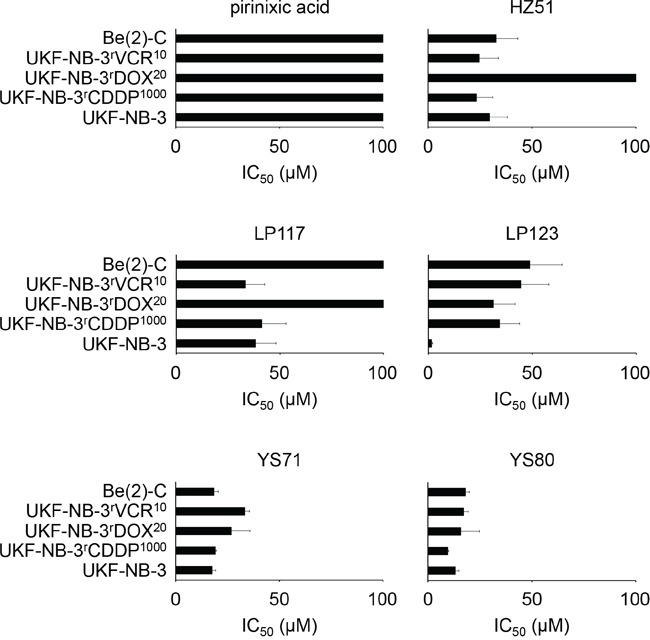
Effects of pirinixic acid and selected derivatives on the viability of the parental, chemosensitive UKF-NB-3 neuroblastoma cell line, cisplatin- (UKF-NB-3^r^CDDP^1000^), doxorubicin- (UKF-NB-3^r^DOX^20^), and vincristine-resistant (UKF-NB-3^r^VCR^10^) UKF-NB-3 sub-lines, and drug-resistant Be(2)-C neuroblastoma cells Concentrations that reduce cell viability by 50% (IC_50_) were determined after 120h of incubation by MTT assay. The corresponding numerical values are presented in Suppl. Table 2.

All cell lines were again insensitive to pirinixic acid in concentrations up to 100μM (Figure 2, Suppl. Table 2). The drug-resistant UKF-NB-3 sub-lines were similarly or less sensitive to HZ51, LP117, LP123, YS71, and YS80 relative to UKF-NB-3. All three drug-resistant UKF-NB-3 sub-lines displayed substantial cross-resistance to LP123. The fold changes IC_50_ resistant UKF-NB-3 sub-line/UKF-NB-3 ranged from 20.19 to 28.66. UKF-NB-3^r^DOX^20^ cells were substantially (>2-fold) more resistant to HZ51 and LP117 than UKF-NB-3 cells. All other fold changes (IC_50_ resistant UKF-NB-3 sub-line/UKF-NB-3) were >0.5 and <2 (Figure 2, Suppl. Table 2). Be(2)-C cells displayed decreased sensitivity to LP117 compared to UKF-NB-3 and similar sensitivity to the other tested compounds (Figure 2, Suppl. Table 2).

### Effects of selected pirinixic acid derivatives on the vincristine sensitivity of vincristine-resistant UKF-NB-3^r^VCR^10^ cells

**Figure 3:**
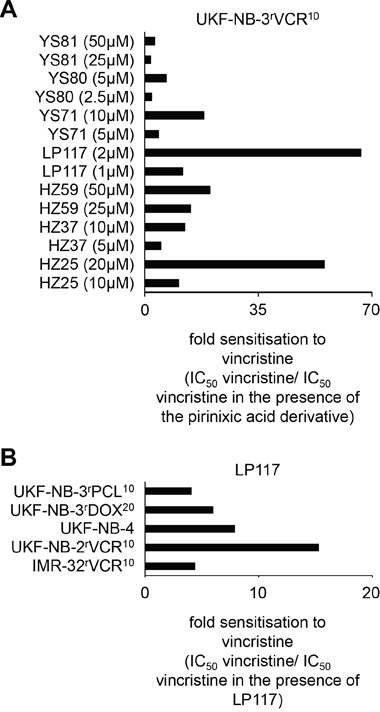
Effects of pirinixic acid derivatives on the sensitivity of ABCB1-expressing cell lines to the cytotoxic ABCB1 substrate vincristine **A.** Fold sensitisation of UKF-NB-3^r^VCR^10^ cells to vincristine by selected pirinixic acid derivatives (IC_50_ vincristine/IC_50_ vincristine in the presence of the pirinixic acid derivative). The corresponding numerical values are presented in Suppl. Table 3. **B.** Fold sensitisation of different ABCB1-expressing cell lines to vincristine by LP117 (2μM) (IC_50_ vincristine/IC_50_ vincristine in the presence of LP117). The corresponding numerical values are presented in Suppl. Table 4.

Next, we investigated the effects of a range of pirinixic acid derivatives that had displayed varying effects on UKF-NB-3 neuroblastoma cell sensitivity (HZ25, IC_50_ 67.64 ± 31.39μM; HZ37, IC_50_ >100μM; HZ59, IC_50_ >100μM; LP117, IC_50_ 29.36 ± 12.42μM; YS71, IC_50_ 26.51 ± 1.94μM; YS80, IC_50_ 9.87 ± 1.19μM; YS81, >100μM, Figure 1, Suppl. Table 1) on the vincristine sensitivity of the vincristine-resistant UKF-NB-3 sub-line UKF-NB-3^r^VCR^10^. In the absence of vincristine, these compounds had exerted similar effects on the viability of UKF-NB-3 and UKF-NB-3^r^VCR^10^ cells (IC_50_ UKF-NB-3^r^VCR^10^/IC_50_ UKF-NB-3 >0.5 and <2): HZ25, IC_50_ >100μM; HZ37, IC_50_ >100μM; HZ59, IC_50_ > 100μM; LP117, IC_50_ 33.39 ± 9.11μM; YS71, IC_50_ 33.25 ± 2.30μM; YS80, IC_50_ 17.16 ± 0.80μM; YS81, >100μM (Figure 2, Suppl. Table 2). All tested pirinixic acid derivatives sensitized UKF-NB-3^r^VCR^10^ cells to vincristine (Figure 3A, Suppl. Table 3).

### Effects of LP117 on the sensitivity of ABCB1-expressing cells to ABCB1 substrates

UKF-NB-3^r^VCR^10^ cells express high ABCB1 levels, and ABCB1 inhibitors sensitize UKF-NB-3^r^VCR^10^ cells to the ABCB1 substrate vincristine [20,21]. LP117 had reduced the vincristine IC_50_ in UKF-NB-3^r^VCR^10^ cells in low micromolar concentrations (Figure 3A, Suppl. Table 3). LP117 (2μM) also sensitized the vincristine-resistant ABCB1-expressing neuroblastoma cell lines IMR-32^r^VCR^10^ and UKF-NB-2^r^VCR^10^, the ABCB1-expressing neuroblastoma cell line UKF-NB-4 that was isolated as multi-drug resistant cell line from a patient [19,20,22], the ABCB1-expressing doxorubicin-resistant UKF-NB-3 sub-line UKF-NB-3^r^DOX^20^, and the ABCB1-expressing paclitaxel-resistant UKF-NB-3 sub-line UKF-NB-3^r^PCL^10^ to vincristine (Figure 3B, Suppl. Table 4). In contrast, LP117 did neither sensitize non-ABCB1-expressing UKF-NB-3 cells to vincristine (Suppl. Table 4) nor ABCB1-expressing UKF-NB-3^r^VCR^10^ or UKF-NB-3^r^DOX^20^ cells to the non-ABCB1 substrate cisplatin (Suppl. Table 5).

**Figure 4:**
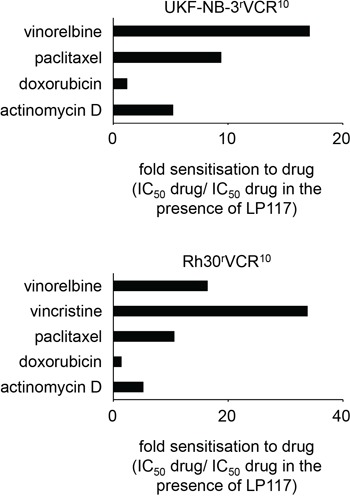
Effects of LP117 on the sensitivity of the ABCB1-expressing neuroblastoma cell line UKF-NB-3^r^VCR^10^ and the ABCB1-expressing rhabdhomyosarcoma cell line Rh30^r^VCR^10^ to different cytotoxic ABCB1 substrates Values are presented as fold sensitization (IC_50_ ABCB1 substrate/IC_50_ ABCB1 substrate in the presence of LP117). The corresponding numerical values are presented in Suppl. Table 6 and 7.

**Figure 5:**
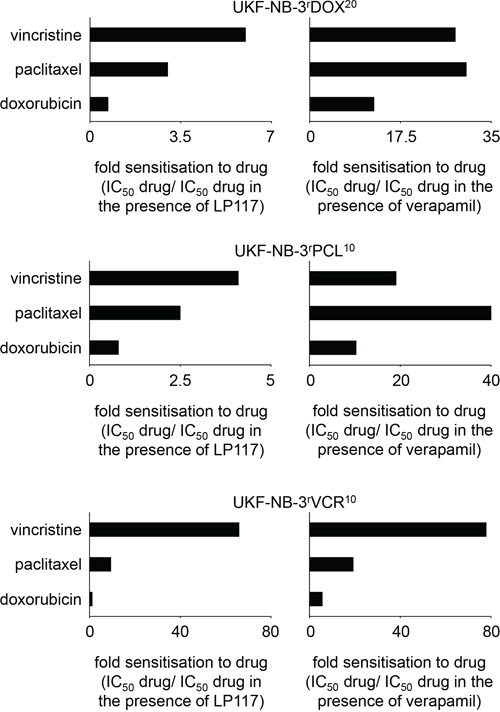
Effects of LP117 or the ABCB1 inhibitor verapamil on the sensitivity of the ABCB1-expressing neuroblastoma cell lines UKF-NB-3^r^DOX^20^, UKF-NB-3^r^PCL^10^, or UKF-NB-3^r^VCR^10^ to different cytotoxic ABCB1 substrates Values are presented as fold sensitization (IC_50_ ABCB1 substrate/IC_50_ ABCB1 substrate in the presence of LP117 or verapamil, respectively). The corresponding numerical values are presented in Suppl. Table 8.

LP117 further sensitized UKF-NB-3^r^VCR^10^ cells to the ABCB1 substrates vinorelbine, paclitaxel, and actinomycin D but not to the ABCB1 substrate doxorubicin in the observed concentration range of up to 2μM (Figure 4, Suppl. Table 6). Similar results were obtained in the vincristine-resistant ABCB1-expressing rhabdomyosarcoma cell line Rh30^r^VCR^10^ (Figure 4, Suppl. Table 7). These data suggest that LP117 predominantly interferes with the ABCB1-mediated transport of certain ABCB1 substrates. In concordance, LP117 sensitized UKF-NB-3^r^DOX^20^ and UKF-NB-3^r^PCL^10^ cells to vincristine and paclitaxel but not to doxorubicin (Figure 5, Suppl. Table 8). The known ABCB1 inhibitor verapamil sensitized UKF-NB-3^r^DOX^20^, UKF-NB-3^r^PCL^10^, and UKF-NB-3^r^VCR^10^ cells to all three drugs although it exerted much more pronounced effects on vincristine and paclitaxel than on doxorubicin (Figure 5, Suppl. Table 8).

**Figure 6:**
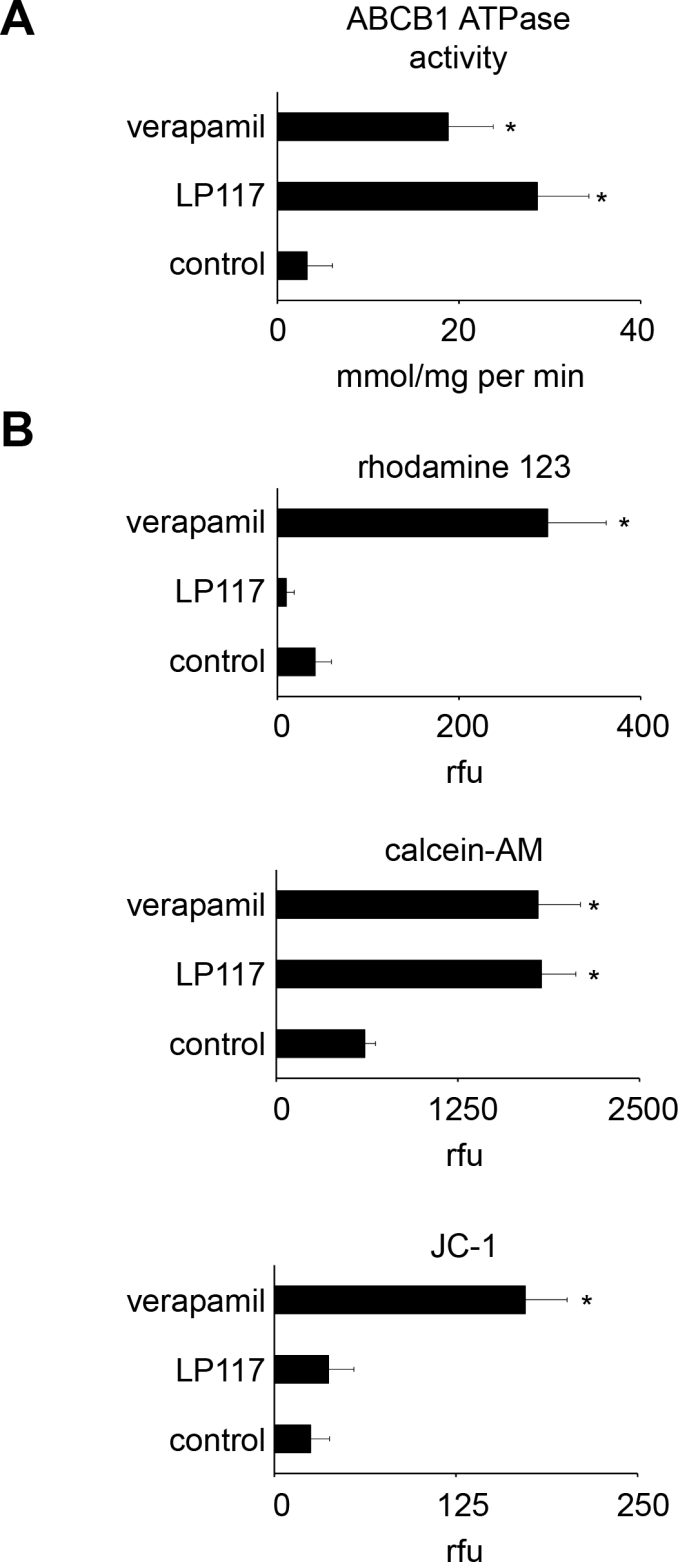
Effects of LP117 and the ABCB1 substrate verapamil on ABCB1 ATPase activity in isolated membranes and the accumulation of fluorescent ABCB1 substrates in ABCB1-expressing UKF-NB-3^r^VCR^10^ cells **A.** ABCB1 ATPase activity in the absence or presence of LP117 (2μM) or verapamil (5μM) as determined in an isolated membrane assay, **B.** Accumulation of the fluorescent ABCB1 substrates rhodamine 123 (0,5μM), calcein-AM (1μM), or JC-1 (0.1μM) in the absence or presence of LP117 (2μM) or verapamil (5μM). * P < 0.05 relative to untreated control.

Both LP117 and verapamil induced ABCB1-ATPase activity in isolated membranes (Figure 6A). However, only verapamil but not LP117 caused accumulation of the fluorescent ABCB1 substrate rhodamine 123 in UKF-NB-3^r^VCR^10^ cells in the investigated concentrations between 2 and 10μM (Figure 6B; Suppl. Figure 1). Furthermore, both compounds enhanced accumulation of the fluorescent ABCB1 substrate calcein-AM in UKF-NB-3^r^VCR^10^ cells but only verapamil enhanced accumulation of JC-1, another fluorescent ABCB1 substrate (Figure 6B).

The specific interaction with the ABCB1-mediated efflux of a limited number of substrates appears to be a common feature of the investigated pirinixic acid derivatives since none of the other pirinixic acid derivatives that sensitized UKF-NB-3^r^VCR^10^ cells to vincristine (HZ25, HZ37, HZ59, YS71, YS80) influenced rhodamine 123 accumulation in UKF-NB-3^r^VCR^10^ cells in the investigated concentrations (Suppl. Table 9).

### Docking experiments

**Figure 7:**
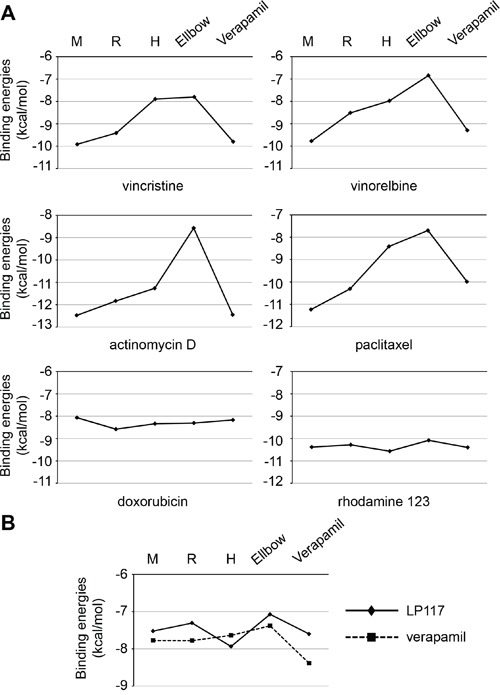
Interaction profiles of ABCB1 substrates with ABCB1 binding sites as indicated by the binding energies of the top pose (ΔG) in kcal/mol derived from *in silico* docking studies **A.** Profiles of cytotoxic and fluorescent ABCB1 substrates; **B.** Direct comparison of LP117 (diamonds) and verapamil (squares, dotted line). The corresponding numerical values are presented in Suppl. Table 10.

We performed docking experiments to gain further insights into the mechanisms underlying the interaction of pirinixic acid derivatives with ABCB1. The results revealed complex interaction profiles of the investigated compounds with the different putative ABCB1 binding sites (Suppl. Table 10). LP117 had sensitized ABCB1-expressing cells to vincristine, vinorelbine, paclitaxel, and actinomycin D but not to doxorubicin (Figure 4). Both, LP117 and verapamil had stronger sensitized ABCB1-expressing cells to vincristine and paclitaxel than to doxorubicin (Figure 5). Indeed, the ABCB1-binding site interaction profiles of vincristine, vinorelbine, paclitaxel, and actinomycin D displayed similarities but clearly differed from the profile of doxorubicin (Figure 7A). Moreover, LP117 and verapamil had displayed differing effects on rhodamine 123 accumulation in ABCB1-expressing cells. Verapamil caused rhodamine 123 accumulation in UKF-NB-3^r^VCR^10^ cells, while LP117 did not modify cellular rhodamine 123 levels in the investigated concentrations of up to 10μM (Figure 6; Suppl. Figure 1). In accordance, the docking studies indicated differences in the ABCB1 binding site interaction profiles of LP117 and verapamil that may contribute to this discrepancy (Figure 7B). These differences may also play a role with regard to the disparities in the relative effects on vincristine and paclitaxel efficacy that were observed between LP117 and verapamil in the ABCB1-expressing cell lines UKF-NB-3^r^DOX^20^, UKF-NB-3^r^PCL^10^, and UKF-NB-3^r^VCR^10^ (Figure 5). LP117 displayed stronger effects on vincristine activity than on paclitaxel activity in all three cell lines. In contrast, verapamil exerted similar effects on vincristine and paclitaxel in UKF-NB-3^r^DOX^20^ cells, more pronounced effects on paclitaxel than on vincristine in UKF-NB-3^r^PCL^10^ cells, and more pronounced effects on vincristine than on paclitaxel in UKF-NB-3^r^VCR^10^ cells (Figure 5). Notably, these cell line-dependent effects of verapamil on vincristine and paclitaxel efficacy may point to differences in the composition of the cell membrane that may influence transporter activity [23]. In addition, discordances in ABCB1 sequence and/or structure between the cell lines may contribute to this phenomenon.

## DISCUSSION

In this study, we screened a panel of pirinixic acid derivatives for effects on the viability of the neuroblastoma cell line UKF-NB-3 and the prostate carcinoma cell line PC-3. HZ53 (IC_50_ UKF-NB-3: 4.66 ± 1.85, IC_50_ PC-3: 10.76 ± 0.23), LP123 (IC_50_ UKF-NB-3: 2.04 ± 0.69, IC_50_ PC-3: 58.23 ± 17.65), and YS80 (IC_50_ UKF-NB-3: 9.87 ± 1.19, IC_50_ PC-3: 11.61 ± 7.83) were the only compounds that displayed IC_50_ values below 10μM in at least one of the cell lines. Further research will have to show whether some of these compounds may exert promising anti-cancer activity in a broader range of models.

The investigated pirinixic acid derivatives modulate 5-LO, mPGES1, PPARα, and PPARγ at varying potencies [3–8] and these molecules are considered as potential anti-cancer drug targets [14–17]. However, the effects of the compounds on cancer cell viability did not correlate with their effects on 5-LO, mPGES1, PPARα, or PPARγ activity (Suppl. Table 11) suggesting that interference with these molecules is not critical for their anti-cancer activity in the investigated cell lines.

HZ51, LP117, LP123, YS71, and YS80 were characterized by varying activity profiles in a set of drug-resistant neuroblastoma cell lines consisting of the cisplatin- (UKF-NB-3^r^CDDP^1000^), doxorubicin- (UKF-NB-3^r^DOX^20^), and vincristine-resistant (UKF-NB-3^r^VCR^10^) sub-lines of UKF-NB-3 [21,24] and the cell line Be(2)-C, a multi-drug resistant neuroblastoma cell line that was established from a patient post-treatment [18–20]. YS71 and YS80 displayed similar efficacy in the resistant neuroblastoma cell lines compared to UKF-NB-3 as indicated by fold changes IC_50_ resistant cell line/IC_50_ UKF-NB-3 < 2. All four resistant cell lines showed enhanced resistance to LP123 as indicated by fold changes IC_50_ resistant cell line/IC_50_ UKF-NB-3 > 2. HZ51 was similarly effective to UKF-NB-3 in all resistant cell lines but UKF-NB-3^r^DOX^20^, while UKF-NB-3^r^DOX^20^ and Be(2)-C cells were resistant to LP117. These findings suggest that the investigated pirinixic acid derivatives differ in the mechanisms underlying their effects on cancer cell viability.

A range of pirinixic acid derivatives sensitized ABCB1-expressing UKF-NB-3^r^VCR^10^ cells to vincristine, with LP117 being the most potent one. LP117 also sensitized a range of further ABCB1-expressing cell lines to vincristine, including two other neuroblastoma cell lines with acquired resistance to vincristine (IMR-32^r^VCR^10^, UKF-NB-2^r^VCR^10^), UKF-NB-3 sub-lines adapted to doxorubicin (UKF-NB-3^r^DOX^20^) or paclitaxel (UKF-NB-3^r^PCL^10^), and the intrinsically ABCB1-expressing neuroblastoma cell line UKF-NB-4 [20,22,24] but did not sensitize UKF-NB-3^r^DOX^20^ or UKF-NB-3^r^VCR^10^ cells to the non-ABCB1 substrate cisplatin. These data, together with the finding that LP117 stimulates ABCB1 ATPase activity, suggest that LP117 and other pirinixic derivatives interfere with ABCB1 function, possibly being substrates.

Most strikingly, LP117 sensitized ABCB1-expressing cells to ABCB1 substrates including vincristine, vinorelbine, actinomycin D, and paclitaxel but not to doxorubicin. Among the three fluorescent ABCB1 substrates, LP117 only caused cellular accumulation of calcein-AM but not of rhodamine 123 or JC-1 in the investigated concentration range. This suggests that LP117 interferes with ABCB1-mediated drug transport in a substrate-specific fashion. Such a substrate-specific interaction with ABCB1 function appears to be a common feature of the investigated pirinixic acid derivatives since HZ25, HZ37, HZ59, YS71, and YS80 all sensitized ABCB1-expressing cells to vincristine but did not cause rhodamine 123 accumulation in ABCB1-expressing cells. Verapamil also displayed stronger effects on the sensitivity of ABCB1-expressing cells to vincristine and paclitaxel than to doxorubicin. Indeed, *in silico* docking studies indicated a notable difference in the interaction profile of doxorubicin with known ABCB1 binding sites compared to vincristine, vinorelbine, paclitaxel, and actinomycin D that may contribute to the discrepancies observed. LP117 and verapamil differed in their interactions with rhodamine 123. Only verapamil caused rhodamine 123 accumulation in ABCB1-expressing cells in the tested concentration range of up to 10μM. In accordance, *in silico* docking studies revealed recognizable disparities in the ABCB1 binding site interaction profiles of LP117 and verapamil.

Our data shed further light on the complexity of substrate interactions with ABCB1 adding to previous reports that had suggested that the mode and/or strength of ABCB1 interaction may differ among ABCB1 substrates [25–30]. Notably, the composition of the cell membrane and ABCB1 polymorphisms/mutations are also known to modulate ABCB1 function and substrate specificity [23;31]. In this context, we here showed that the relative effects of verapamil on paclitaxel and vincristine toxicity differed between the ABCB1-expressing cell lines UKF-NB-3^r^DOX^20^ (doxorubicin-resistant), UKF-NB-3^r^PCL^10^ (paclitaxel-resistant), and UKF-NB-3^r^VCR^10^ (vincristine-resistant). Verapamil sensitized UKF-NB-3^r^DOX^20^ cells to paclitaxel and vincristine in a similar fashion but displayed more pronounced effects on paclitaxel in UKF-NB-3^r^PCL^10^ cells and more pronounced effects on vincristine in UKF-NB-3^r^VCR^10^ cells. This may indicate that cancer cell adaptation to ABCB1 substrates may not only result in increased ABCB1 expression but potentially also to further changes that increase the specificity of ABCB1 to certain substrates.

More targeted approaches combining pharmacologically active ABCB1 substrates with tailored ABCB1 inhibitors would offer novel therapeutic opportunities. High ABCB1 levels represent an important cancer cell resistance mechanism [32–34]. However, ABCB1 inhibitors failed in a number of clinical cancer trials [32,33]. One of the reasons for these failures is that ABCB1 inhibitors did not only interfere with the ABCB1 present on cancer cells but also with ABCB1 at physiological cellular and tissue barriers. Thus, ABCB1 inhibitors affected the adsorption and distribution of concomitantly administered drugs (and possibly xenobiotics) resulting in toxicity and unpredictable pharmacokinetics [32,33]. Tailored combinations of drugs that are ABCB1 substrates and respective drug-specific ABCB1 inhibitors may help to overcome this problem. In addition, such specific ABCB1 substrate/ABCB1 inhibitor combinations may also improve the oral bioavailability of drugs and drug penetration into the central nervous system [32,33].

In conclusion, we provide evidence that pirinixic acid derivatives interfere with ABCB1-mediated drug transport in a substrate-specific manner.

## MATERIALS AND METHODS

### Drugs

The structures of the investigated compounds are shown in Suppl. Table 12. The compounds were synthesized as previously described [3–8].

### Cells

The prostate carcinoma cell line PC-3 was obtained from DSMZ (Braunschweig, Germany) and the MYCN-amplified neuroblastoma cell lines Be(2)-C and IMR-32 from ATCC (Manassas, VA, USA). The alveolar rhabdomyosarcoma cell line Rh30 was kindly provided by Dr. P.J. Houghton (St. Jude's Children's Research Hospital, Memphis, Tennessee). The MYCN-amplified neuroblastoma cell lines UKF-NB-2, UKF-NB-3, and UKF-NB-4 were established from stage 4 neuroblastoma patients [21,22,35].

The following drug-adapted cell lines were derived from the resistant cancer cell line (RCCL) collection (http://www.kent.ac.uk/stms/cmp/RCCL/RCCLabout.html): IMR-32^r^VCR^10^ (vincristine), UKF-NB-2^r^VCR^10^, UKF-NB-3^r^CDDP^1000^ (cisplatin), UKF-NB-3^r^DOX^20^ (doxorubicin), UKF-NB-3^r^PCL^10^ (paclitaxel), UKF-NB-3^r^VCR^10^, Rh30^r^VCR^10^. Parental chemosensitive cell lines had been adapted to growth in the presence of anti-cancer drugs by continuous exposure to increasing drug concentrations as described previously [21,35,36].

All cells were propagated at 37°C in IMDM supplemented with 10 % FBS, 100 IU/ml penicillin, and 100 μg/ml streptomycin. Cells were routinely tested for mycoplasma contamination and authenticated by short tandem repeat profiling.

### Viability assay

Cell viability was tested by the 3-(4,5-dimethylthiazol-2-yl)-2,5-diphenyltetrazolium bromide (MTT) dye reduction assay after 120 h incubation modified as previously described [21,24,35,36].

### Determination of ABCB1 ATPase activity

The ABCB1-ATPase activity was determined using membrane preparations (BD Biosciences, Heidelberg, Germany) and an established kit (BD Biosciences, Heidelberg, Germany) following the manufacturer's instruction.

### Flow cytometry

The ABCB1-mediated efflux of fluorescent ABCB1 substrates was determined as described previously [20]. Cells were incubated with rhodamine 123 (0.5μM), JC-1 (0.1μM), or calcein-AM (1μM) for 60 min. Then cells were washed with PBS and incubated for another 60 min to allow ABCB1-mediated drug efflux. Subsequently, cellular fluorescence was analyzed by flow cytometry (FACSCalibur, Becton Dickinson, Heidelberg, Germany). Rhodamine 123 was detected at the FL1 channel. JC-1 and calcein-AM were detected at the FL2 channel.

### Docking experiments

Molecular docking experiments were performed using the most recently solved crystal structure of murine holo Abcb1 (PDB code: 4Q9J), the holo structure with the highest resolution to date (3.6 Å), complexed with three cyclic peptide structures (QZ-Val), one of which is located at a possibly new binding site at the elbow helix-2 region [37]. All docking was carried out using MOE (version 2013.08, Chemical Computing Group Inc., Montreal, Canada). The protein structure provided in the PDB file was edited using the *Protonate3D* protocol in MOE which consisted of capping termini and chain breaks, and protonation and charge correction using AMBER99 for charge assignment. The structure was minimized using the *ligX* protocol under “tethered” conditions to avoid any considerable displacements from the original crystal structure. The optimized structure was used first to validate our docking protocol by re-docking the co-crystallized ligand (QZ-Val) at the three respective locations in the co-crystallized structure. This exercise resulted in very low Root-mean-square deviation (RMSD) values between the docked and the co-crystallized structures (RMSD < 1Å), which indicates the ability of achieving a near-native ligand pose. All the compounds used in the cell sensitization experiments were docked into the binding sites of the Abcb1 structure. All ligands were minimized using self-consistent field (SCF) method with PM6 Hamiltonian for partial charge assignment. The compounds were then docked at several different binding sites suggested by the literature for substrate or ATP binding. This included the M-site, R-site, H-site, elbow helix-2, verapamil, and the ATP 1 and ATP 2 binding sites. The amino acid residues defining these binding sites are presented in Suppl. Table 13 and their positions are shown in Suppl. Figure 2. A flexible docking protocol was used where the amino acid residues in the binding site were allowed a constrained movement. After this flexible docking the top 60 London dG scoring poses were kept, then re-docked and re-scored using GBVI/WSA dG scoring in MOE and the top pose was used in the analysis.

### Statistics

Results are expressed as mean ± S.D. of at least three experiments. Comparisons between two groups were performed using Student's t-test. Three and more groups were compared by ANOVA followed by the Student-Newman-Keuls test. P values lower than 0.05 were considered to be significant.

## SUPPLEMENTARY DATA FIGURES AND TABLES






