# Supplementary material for: Substrate-specific effects of pirinixic acid derivatives on ABCB1-mediated drug transport
Source: Oncotarget. 2016 Feb 12;7(10):11664–76. doi: 10.18632/oncotarget.7345 (PMC4905501; doi:10.18632/oncotarget.7345)
Supplement: Supplementary file 1 [file oncotarget-07-11664-s001.pdf]

SUPPLEMENTARY DATA

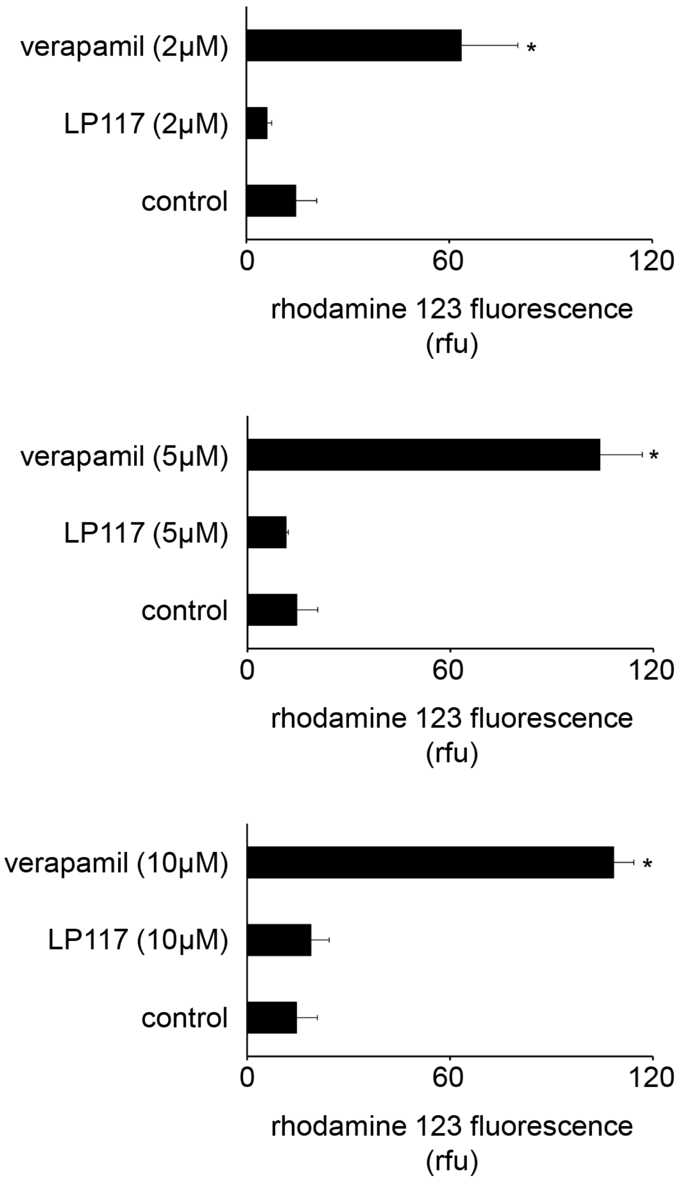

Supplementary Figure S1: Effects of varying LP117 and verapamil concentrations on the accumulation of the ABCB1 substrate rhodamine 123 in ABCB1-expressing UKF-NB-3rVCR10 cells as determined by flow cytometry (rfu = relative fluorescence units). * $P < 0.05$ relative to control.

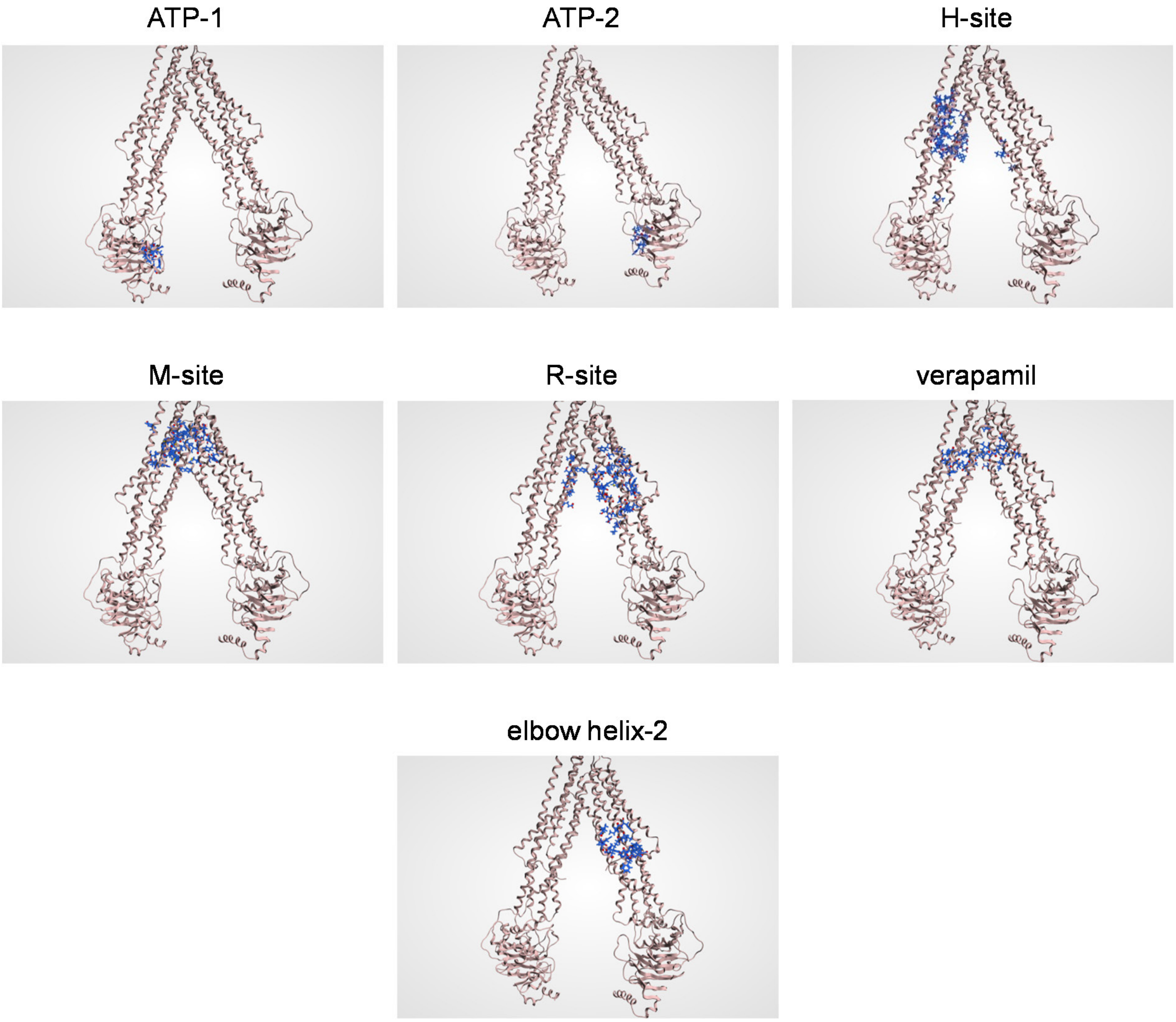

Supplementary Figure S2: Crystal structure of Abcb1. Binding site residues are highlighted.

Supplementary Table S1: Effects of pirinixic acid and its derivatives on UKF-NB-3 neuroblastoma and PC-3 prostate cancer cell viability

	UKF-NB-3	PC-3
**Compound**	**IC_{50} (μM)**	**IC_{50} (μM)**
pirinixic acid	> 100[1]	> 100
HZ18	40.38 ± 0.88[2]	79.46 ± 1.89
HZ20	> 100	> 100
HZ25	67.64 ± 31.39	> 100
HZ27	> 100	> 100
HZ28	> 100	> 100
HZ34	> 100	87.84 ± 6.43
HZ37	> 100	17.97 ± 5.62
HZ42	57.98 ± 10.21	86.37 ± 4.14
HZ47	> 100	> 100
HZ49	13.25 ± 4.89	> 100
HZ51	25.42 ± 6.61	> 100
HZ52	83.88 ± 15.63	79.21 ± 1.87
HZ53	4.66 ± 1.85	10.76 ± 0.23
HZ55	> 100	71.23 ± 12.99
HZ56	69.71 ± 0.76	41.12 ± 0.71
HZ59	> 100	> 100
HZ61	> 100	> 100
HZ64	> 100	> 100
HZ65	70.71 ± 10.00	64.00 ± 7.26
HZ74	> 100	21.50 ± 6.04
HZ75	84.49 ± 15.41	75.48 ± 3.46
HZ76	> 100	> 100
HZ82	> 100	> 100
HZ97	> 100	> 100
LP105	50.75 ± 1.33	42.12 ± 12.40
LP117	29.36 ± 12.42	16.14 ± 0.74
LP119	> 100	31.06 ± 5.85
LP120	51.54 ± 2.78	65.52 ± 11.89
LP121	> 100	> 100
LP123	2.04 ± 0.69	58.23 ± 17.65
YS49	> 100	> 100
YS71	26.51 ± 1.94	50.18 ± 0.30
YS78	> 100	> 100
YS80	9.87 ± 1.19	11.61 ± 7.83
YS81	> 100	> 100
YS82	> 100	> 100
YS83	> 100	> 100
YS85	86.23 ± 13.61	71.04 ± 25.43
YS121	> 100	> 100

IC_{50} values determined after 120h of incubation by MTT assay.
[1] 100μM was the highest concentration tested
[2] values are mean ± S.D. of at least three experiments

Supplementary Table S2: Effects of pirinixic acid and selected derivatives on the viability of the parental, chemosensitive UKF-NB-3 neuroblastoma cell line, cisplatin- (UKF-NB-3^{r}CDDP^{1000}), doxorubicin- (UKF-NB-3^{r}DOX^{20}), and vincristine-resistant (UKF-NB-3^{r}VCR^{10}) UKF-NB-3 sub-lines, and drug-resistant Be(2)-C neuroblastoma cells

	IC$_{50}$ (µM)					
**Cell line**	**pirinixic acid**	**HZ51**	**LP117**	**LP123**	**YS71**	**YS80**
UKF-NB-3	> 100	29.64 ± 8.49	38.14 ± 9.83	1.55 ± 0.49	17.48 ± 1.85	13.10 ± 6.38
UKF-NB-3^{r}CDDP^{1000}	> 100	23.24 ± 7.91 (0.78)[1]	41.18 ± 11.62 (1.08)	34.20 ± 9.65 (22.06)	19.18 ± 0.42 (1.10)	9.62 ± 0.36 (0.73)
UKF-NB-3^{r}DOX^{20}	> 100	> 100 (>3.37)	> 100 (>2.62)	31.30 ± 10.41 (20.19)	26.77 ± 9.07 (1.53)	15.81 ± 4.03 (1.21)
UKF-NB-3^{r}VCR^{10}	> 100	24.69 ± 9.07 (0.83)	33.39 ± 9.11 (0.88)	44.42 ± 13.48 (28.66)	33.25 ± 2.30 (1.90)	17.16 ± 0.80 (1.31)
Be(2)-C	> 100	32.73 ± 10.42 (1.10)[2]	> 100 (>2.62)	48.78 ± 15.52 (31.47)	18.52 ± 1.99 (1.06)	18.02 ± 2.26 (1.38)

Concentrations that reduce cell viability by 50% (IC$_{50}$) were determined after 120h of incubation by MTT assay.
[1] fold change (IC$_{50}$ resistant UKF-NB-3 sub-line/ IC$_{50}$ UKF-NB-3)
[2] fold change (IC$_{50}$ resistant Be(2)-C/ IC$_{50}$ UKF-NB-3)

Supplementary Table S3: Effects of selected pirinixic acid derivatives on the sensitivity of vincristine-resistant UKF-NB-3[r]VCR[10] cells to vincristine

	pirinixic acid derivative alone (% control)	IC_{50} vincristine (ng/mL) vincristine alone	+ pirinixic acid derivative	fold change[1]
HZ25 10µM	91 ± 15	68.68 ± 7.17	6.54 ± 2.01	10.5
HZ25 20µM	86 ± 20	68.68 ± 7.17	1.24 ± 0.33	55.4
HZ37 5µM	92 ± 10	68.68 ± 7.17	13.87 ± 4.16	5.0
HZ37 10µM	83 ± 19	68.68 ± 7.17	5.52 ± 2.12	12.4
HZ59 25µM	98 ± 18	68.68 ± 7.17	4.84 ± 1.90	14.2
HZ59 50µM	93 ± 9	68.68 ± 7.17	3.39 ± 1.45	20.3
LP117 1µM	92 ± 13	68.68 ± 7.17	5.85 ± 1.87	11.7
LP117 2µM	95 ± 14	68.68 ± 7.17	1.03 ± 0.42	66.7
YS71 5µM	96 ± 7	68.68 ± 7.17	16.00 ± 4.92	4.3
YS71 10µM	95 ± 11	68.68 ± 7.17	3.75 ± 0.88	18.3
YS80 2.5µM	108 ± 7	68.68 ± 7.17	31.72 ± 9.58	2.2
YS80 5µM	99 ± 16	68.68 ± 7.17	10.30 ± 2.47	6.7
YS81 25µM	91 ± 6	68.68 ± 7.17	36.68 ± 11.84	1.9
YS81 50µM	68 ± 14	68.68 ± 7.17	22.17 ± 6.63	3.1

Cell viability and concentrations that reduce cell viability by 50% (IC_{50}) were determined after 120h of incubation by MTT assay.

[1] fold change (vincristine IC_{50} / vincristine IC_{50} in the presence of the respective pirinixic acid derivative)

Supplementary Table S4: Effects of LP117 (2µM) on the sensitivity of cell lines with high or low ABCB1 expression to the cytotoxic ABCB1 substrate vincristine

cell line	viability in the presence of LP117 alone (% control)	IC_{50} vincristine (ng/mL)		fold change[1]
		vincristine alone	+ LP117	
IMR-32^{r}VCR^{10}	92 ± 18	24.76 ± 4.20	5.62 ± 0.70	4.4
UKF-NB-2^{r}VCR^{10}	76 ± 22	60.72 ± 24.46	3.97 ± 0.29	15.3
UKF-NB-4	95 ± 17	33.43 ± 5.13	4.23 ± 0.90	7.9
UKF-NB-3^{r}DOX^{20}	93 ± 12	18.85 ± 3.84	3.13 ± 0.96	6.0
UKF-NB-3^{r}PCL^{10}	89 ± 10	20.36 ± 4.91	4.91 ± 1.62	4.1
UKF-NB-3	92 ± 13	0.25 ± 0.08	0.21 ± 0.07	1.2

Cell viability and concentrations that reduce cell viability by 50% (IC_{50}) were determined after 120h of incubation by MTT assay.

[1] fold change (vincristine IC_{50} / vincristine IC_{50} in the presence of LP117)

Supplementary Table S5: Effects of LP117 (2µM) on the sensitivity of cell lines with high ABCB1 expression to the cytotoxic non-ABCB1 substrate cisplatin

cell line	viability in the presence of LP117 alone (% control)	IC_{50} cisplatin (ng/mL)		fold change[1]
		cisplatin alone	+ LP117	
UKF-NB-3^{r}VCR^{10}	100 ± 12	453 ± 107	505 ± 139	0.9
UKF-NB-3^{r}DOX^{20}	89 ± 11	276 ± 87	241 ± 70	1.1

Cell viability and concentrations that reduce cell viability by 50% (IC_{50}) were determined after 120h of incubation by MTT assay.

[1] fold change (cisplatin IC_{50} / cisplatin IC_{50} in the presence of LP117)

Supplementary Table S6: Effects of LP117 (2µM) on the sensitivity of ABCB1-expressing UKF-NB-3^{r}VCR^{10} cells to various cytotoxic ABCB1 substrates

drug	viability in the presence of LP117 alone (% control)	IC_{50} (ng/mL)		fold change[1]
		ABCB1 substrate alone	+ LP117	
actinomycin D	100 ± 12	7.18 ± 1.46	1.38 ± 0.27	5.2
doxorubicin	100 ± 12	41.33 ± 1.31	35.34 ± 14.14	1.2
paclitaxel	100 ± 12	54.48 ± 16.47	5.77 ± 1.86	9.4
vinorelbine	100 ± 12	71.77 ± 29.96	4.19 ± 1.18	17.1

Cell viability and concentrations that reduce cell viability by 50% (IC_{50}) were determined after 120h of incubation by MTT assay.

[1] fold change (IC_{50} ABCB1 substrate/ IC_{50} ABCB1 substrate in the presence of LP117)

Supplementary Table S7: Effects of LP117 (2µM) on the sensitivity of ABCB1-expressing Rh30[r]VCR[10] cells to various cytotoxic ABCB1 substrates

drug	viability in the presence of LP117 alone (% control)	IC_{50} (ng/mL)		fold change[1]
		ABCB1 substrate alone	+ LP117	
actinomycin D	98 ± 10	4.95 ± 1.09	0.96 ± 0.24	5.2
doxorubicin	98 ± 10	41.99 ± 9.28	28.97 ± 7.31	1.4
paclitaxel	98 ± 10	54.37 ± 16.03	5.12 ± 1.58	10.6
vincristine	98 ± 10	38.55 ± 6.86	1.14 ± 0.25	33.8
vinorelbine	98 ± 10	45.73 ± 13.62	2.78 ± 0.71	16.4

Cell viability and concentrations that reduce cell viability by 50% (IC_{50}) were determined after 120h of incubation by MTT assay.

[1] fold change (IC_{50} ABCB1 substrate/ IC_{50} ABCB1 substrate in the presence of LP117)

Supplementary Table S8: Effects of LP117 (2µM) or the known ABCB1 inhibitor verapamil (5µM) on the sensitivity of ABCB1-expressing UKF-NB-3^{r}DOX^{20}, UKF-NB-3^{r}PCL^{10}, and UKF-NB-3^{r}VCR^{10} cells to the cytotoxic ABCB1 substrates doxorubicin, paclitaxel, and vincristine. Cell viability and concentrations that reduce cell viability by 50% (IC_{50}) were determined after 120h of incubation by MTT assay.

See Supplementary File S1

Supplementary Table S9: Effects of verapamil or pirinixic acid derivatives on the accumulation of the fluorescent ABCB1 substrate rhodamine 123 (0.5µM) in non-ABCB1-expressing UKF-NB-3 cells and ABCB1-expressing UKF-NB-3^{r}VCR^{10} cells as determined by flow cytometry

	rhodamin 123 fluorescence (rfu)	
Treatment	UKF-NB-3	UKF-NB-3^{r}VCR^{10}
non-treated	2.62 ± 1.04	3.58 ± 1.92
rhodamine 123	1886 ± 375	36.4 ± 9.1
+ verapamil	2473 ± 627	348.3 ± 69.0
HZ25 (10µM)	2637 ± 525	16.2 ± 8.0
HZ37 (10µM)	1959 ± 483	17.3 ± 7.4
HZ59 (25µM)	2485 ± 469	31.3 ± 10.5
LP117 (2µM)	1569 ± 398	14.7 ± 12.4
YS71 (10µM)	2148 ± 531	24.6 ± 8.8
YS80 (5µM)	2091 ± 479	30.4 ± 7.8

Supplementary Table S10: Binding energies of the top pose (ΔG) in kcal/mol calculated at different ABCB1 binding sites

	Substrate binding sites					ATP binding sites	
**Ligands**	**M-site**	**R-site**	**H-site**	**Elbow Helix-2 site**	**Verapamil**	**ATP1**	**ATP2**
HZ51	-7.803	-7.293	-7.095	-6.892	-7.678	-6.221	-6.601
LP123	-6.664	-6.506	-6.075	-6.517	-6.914	-5.289	-5.528
YS71	-6.651	-6.030	-6.213	-6.263	-6.257	-4.937	-5.705
YS80	-7.657	-6.968	-6.308	-6.598	-7.704	-5.998	-6.454
YS81	-7.026	-6.703	-6.060	-6.120	-6.716	-5.388	-5.771
HZ25	-7.680	-7.615	-6.995	-7.406	-7.598	-6.068	-7.122
HZ37	-8.299	-9.458	-6.667	-7.822	-8.069	-6.375	-6.870
HZ59	-7.416	-7.317	-7.933	-5.731	-6.879	-5.773	-6.408
LP117	-7.523	-7.307	-7.935	-7.073	-7.601	-6.655	-6.324
verapamil	-7.774	-7.778	-7.637	-7.381	-8.385	-6.635	-6.869
cisplatin	-3.287	-3.541	-3.307	-3.267	-3.434	-3.090	-3.013
vinorelbine	-9.777	-8.511	-7.975	-6.841	-9.292	-6.965	-7.567
paclitaxel	-11.233	-10.311	-8.415	-7.698	-9.989	-7.291	-8.643
actinomycin D	-12.475	-11.832	-11.263	-8.573	-12.449	-8.429	-9.365
Vincristine	-9.918	-9.411	-7.892	-7.800	-9.802	-6.934	-7.960
Doxorubicin	-8.068	-8.581	-8.339	-8.307	-8.170	-7.001	-7.513
Rhodamine 123	-6.384	-6.281	-6.567	-6.079	-6.399	-5.403	-6.574

Supplementary Table S11: Comparison of the effects of pirinixic acid derivatives on PPARα, PPARγ, 5-LO, and mPGES-1 activity with their effects on the viability of the neuroblastoma cell line UKF-NB-3 and the prostate carcinoma cell line PC-3.

Supplementary Table S11A: Effects of selected pirinixic acid derivatives on PPARα activity and cancer cell viability

Compound	EC_{50} PPARα (μM)[1]	IC_{50}[2] UKF-NB-3 (μM)	IC_{50}[2] PC-3 (μM)
LP121	2.2 (1)[3]	> 100 (3)	> 100 (3)
LP105	10.9 (2)	50.75 (1)	42.12 (1)
LP120	11.5 (3)	51.54 (2)	65.52 (2)
Pirinixic acid	36.3 (4)	> 100 (3)	> 100 (3)

[1] data derived from Popescu L, Rau O, Böttcher J, Syha Y, Schubert-Zsilavecz M. Quinoline-based derivatives of pirinixic acid as dual PPAR alpha/gamma agonists. Arch Pharm (Weinheim) 2007;340(7):367-71.
[2] concentration that reduces cell viability by 50% as determined by MTT assay after 120h of incubation
[3] rank position

Supplementary Table S11B: Effects of selected pirinixic acid derivatives on PPARγ activity and cancer cell viability

Compound	EC_{50} PPARγ (μM)[1]	IC_{50}[2] UKF-NB-3 (μM)	IC_{50}[2] PC-3 (μM)
LP121	3.5 (1)[3]	> 100 (3)	> 100 (3)
LP105	7.5 (2)	50.75 (1)	42.12 (1)
LP120	9.2 (3)	51.54 (2)	65.52 (2)
Pirinixic acid	53.2 (4)	> 100 (3)	> 100 (3)

[1] data derived from Popescu L, Rau O, Böttcher J, Syha Y, Schubert-Zsilavecz M. Quinoline-based derivatives of pirinixic acid as dual PPAR alpha/gamma agonists. Arch Pharm (Weinheim) 2007;340(7):367-71.
[2] concentration that reduces cell viability by 50% as determined by MTT assay after 120h of incubation
[3] rank position

Supplementary Table S11C: Effects of selected pirinixic acid derivatives on PPARα activity and cancer cell viability

Compound	EC_{50} PPARα (μM)[1]	IC_{50}[2] UKF-NB-3 (μM)	IC_{50}[2] PC-3 (μM)
YS85	1.2 (1)[3]	86.23 (1)	71.40 (1)
YS81	7.0 (2)	> 100 (2)	> 100 (2)
YS78	7.7 (3)	> 100 (2)	> 100 (2)
Pirinixic acid	36.3 (4)	> 100 (2)	> 100 (2)

[1] data derived from Rau O, Syha Y, Zettl H, Kock M, Bock A, Schubert-Zsilavecz M. Alpha-alkyl substituted pirinixic acid derivatives as potent dual agonists of the peroxisome proliferator activated receptor alpha and gamma. Arch Pharm (Weinheim) 2008;341(3):191-5.
[2] concentration that reduces cell viability by 50% as determined by MTT assay after 120h of incubation
[3] rank position

Supplementary Table S11D: Effects of selected pirinixic acid derivatives on PPARγ activity and cancer cell viability

Compound	EC_{50} PPARγ (µM)[1]	IC_{50}[2] UKF-NB-3 (µM)	IC_{50}[2] PC-3 (µM)
YS85	3.0 (1)[3]	86.23 (1)	71.40 (1)
YS81	5.5 (2)	> 100 (2)	> 100 (2)
YS78	12.2 (3)	> 100 (2)	> 100 (2)
Pirinixic acid	53.7 (4)	> 100 (2)	> 100 (2)

[1] data derived from Rau O, Syha Y, Zettl H, Kock M, Bock A, Schubert-Zsilavecz M. Alpha-alkyl substituted pirinixic acid derivatives as potent dual agonists of the peroxisome proliferator activated receptor alpha and gamma. Arch Pharm (Weinheim) 2008;341(3):191-5.
[2] concentration that reduces cell viability by 50% as determined by MTT assay after 120h of incubation
[3] rank position

Supplementary Table S11E: Effects of selected pirinixic acid derivatives on 5-LO activity in a whole cell assay using polymorphonuclear leukocytes and on cancer cell viability

Compound	5-LO production at 10µM (% control)[1]	IC_{50}[2] UKF-NB-3 (µM)	IC_{50}[2] PC-3 (µM)
LP117	1.6 (1)[3]	29.36 (4)	16.14 (2)
LP119	4.0 (2)	> 100 (6)	31.06 (3)
YS80	18.7 (3)	9.87 (2)	11.61 (1)
LP121	20.1 (4)	> 100 (6)	> 100 (7)
YS71	22.8 (5)	26.51 (3)	50.18 (4)
YS121	28.9 (6)	> 100 (6)	> 100 (7)
LP120	50.0 (7)	51.54 (5)	65.52 (6)
LP123	77.6 (8)	2.04 (1)	58.23 (5)
YS82	no inhibition (9)	> 100 (6)	> 100 (7)
YS83	no inhibition (9)	> 100 (6)	> 100 (7)

[1] data derived from Werz O, Greiner C, Koeberle A, Hoernig C, George S, Popescu L, Syha I, Schubert-Zsilavecz M, Steinhilber D. Novel and potent inhibitors of 5-lipoxygenase product synthesis based on the structure of pirinixic acid. J Med Chem 2008;51(17):5449-53.
[2] concentration that reduces cell viability by 50% as determined by MTT assay after 120h of incubation
[3] rank position

Supplementary Table S11F: Effects of selected pirinixic acid derivatives on 5-LO activity in a whole cell assay using polymorphonuclear leukocytes and on cancer cell viability

Compound	5-LO production at 10µM (% control)[1]	IC_{50}[2] UKF-NB-3 (µM)	IC_{50}[2] PC-3 (µM)
HZ34	2.7 (1)[3]	> 100 (6)	87.84 (6)
HZ56	2.7 (1)	69.71 (3)	41.12 (2)
HZ42	3.0 (3)	57.98 (1)	86.37 (5)
HZ65	3.1 (4)	70.71 (4)	64.00 (3)
HZ52	4.1 (5)	83.88 (5)	79.21 (4)
HZ47	7.0 (6)	> 100 (6)	> 100 (7)
HZ28	28.0 (7)	> 100 (6)	> 100 (7)
HZ27	40.6 (8)	> 100 (6)	> 100 (7)

(*Continued*)

Compound	5-LO production at 10µM (% control)[1]	IC_{50}[2] UKF-NB-3 (µM)	IC_{50}[2] PC-3 (µM)
HZ20	70.0 (9)	> 100 (6)	> 100 (7)
HZ37	80.2 (10)	> 100 (6)	17.97 (1)
HZ25	80.7 (11)	67.64 (2)	> 100 (7)

[1] data derived from Koeberle A, Zettl H, Greiner C, Wurglics M, Schubert-Zsilavecz M, Werz O. Pirinixic acid derivatives as novel dual inhibitors of microsomal prostaglandin E2 synthase-1 and 5-lipoxygenase. J Med Chem 2008;51(24):8068-76.
[2] concentration that reduces cell viability by 50% as determined by MTT assay after 120h of incubation
[3] rank position

Supplementary Table S11G: Effects of selected pirinixic acid derivatives on mPGES-1 activity and on cancer cell viability

Compound	mPGES-1 activity at 10µM (% control)[1]	IC_{50}[2] UKF-NB-3 (µM)	IC_{50}[2] PC-3 (µM)
HZ20	16.1 (1)[3]	> 100 (6)	> 100 (7)
HZ52	21.7 (2)	83.88 (5)	79.21 (4)
HZ42	21.8 (3)	57.98 (1)	86.37 (5)
HZ65	24.9 (4)	70.71 (4)	64.00 (3)
HZ56	26.1 (5)	69.71 (3)	41.12 (2)
HZ47	29.8 (6)	> 100 (6)	> 100 (7)
HZ25	37.1 (7)	67.64 (2)	> 100 (7)
HZ34	43.1 (8)	> 100 (6)	87.84 (6)
HZ27	66.4 (9)	> 100 (6)	> 100 (7)
HZ28	79.9 (10)	> 100 (6)	> 100 (7)
HZ37	> 100 (11)	> 100 (6)	17.97 (1)

[1] data derived from Koeberle A, Zettl H, Greiner C, Wurglics M, Schubert-Zsilavecz M, Werz O. Pirinixic acid derivatives as novel dual inhibitors of microsomal prostaglandin E2 synthase-1 and 5-lipoxygenase. J Med Chem 2008;51(24):8068-76.
[2] concentration that reduces cell viability by 50% as determined by MTT assay after 120h of incubation
[3] rank position

Supplementary Table S11H: Effects of selected pirinixic acid derivatives on 5-LO activity in a whole cell assay using polymorphonuclear leukocytes and on cancer cell viability

Compound	5-LO production at 10µM (% control)[1]	IC_{50}[2] UKF-NB-3 (µM)	IC_{50}[2] PC-3 (µM)
HZ51	0.8 (1)[3]	25.24 (1)	> 100 (3)
HZ82	1.5 (1)	> 100 (3)	> 100 (3)
HZ75	2.2 (3)	84.49 (2)	75.48 (2)
HZ64	3.0 (4)	> 100 (3)	> 100 (3)
HZ55	> 10 (5)	> 100 (3)	71.23 (1)
HZ76	> 10 (5)	> 100 (3)	> 100 (3)
HZ97	> 10 (5)	> 100 (3)	> 100 (3)

[1] data derived from Greiner C, Zettl H, Koeberle A, Pergola C, Northoff H, Schubert-Zsilavecz M, Werz O. Identification of 2-mercaptohexanoic acids as dual inhibitors of 5-lipoxygenase and microsomal prostaglandin E_2 synthase-1. Bioorg Med Chem 2011;19(11):3394-401.
[2] concentration that reduces cell viability by 50% as determined by MTT assay after 120h of incubation
[3] rank position

Supplementary Table S11I: Effects of selected pirinixic acid derivatives on mPGES-1 activity and on cancer cell viability

Compound	mPGES-1 activity (IC_{50})[1]	IC_{50}[2] UKF-NB-3 (µM)	IC_{50}[2] PC-3 (µM)
HZ82	1.7 (1)[3]	> 100 (2)	> 100 (3)
HZ64	2.9 (2)	> 100 (2)	> 100 (3)
HZ75	3.5 (3)	84.49 (1)	75.48 (2)
HZ55	> 10 (4)	> 100 (2)	71.23 (1)
HZ97	> 10 (5)	> 100 (2)	> 100 (3)

[1] data derived from Greiner C, Zettl H, Koeberle A, Pergola C, Northoff H, Schubert-Zsilavecz M, Werz O. Identification of 2-mercaptohexanoic acids as dual inhibitors of 5-lipoxygenase and microsomal prostaglandin E_2 synthase-1. Bioorg Med Chem 2011;19(11):3394-401.

[2] concentration that reduces cell viability by 50% as determined by MTT assay after 120h of incubation

[3] rank position

Supplementary Table S12: Structures of the investigated compounds

See Supplementary File S2

Supplementary Table S13: Definition of the binding sites that were used for the lining of the docking studies

Mouse P-gp binding site	Residues location	reference
M-site	60, 64, 67, 68, 71, 72, 75, 113, 121, 303, 326, 327, 328, 329, 332, 333, 336, 339, 721, 724, 725, 728, 729, 732, 942, 945, 946, 949, 971, 974, 975, 978, 982	Ferreira et al., 2013
R-site	229, 236, 237, 240, 241, 289, 292, 295, 296, 299, 300, 336, 339, 340, 341, 342, 343, 344, 345, 346, 349, 674, 675, 676, 717, 720, 721, 724, 762, 765, 766, 769, 770, 773, 774, 778, 819, 820, 821, 822, 823, 985, 986, 988, 989, 990, 991, 992, 993	Ferreira et al., 2013
H-site	60, 121, 122, 125, 128, 129, 132, 133, 179, 180, 181, 182, 183, 184, 186, 187, 188, 190, 191, 241, 340, 341, 343, 345, 346, 347, 349, 350, 351, 875, 876, 880, 897, 930, 934, 938, 939, 942, 943, 946, 993, 996	Ferreira et al., 2013
Verapamil	60, 63,64, 335, 121, 218, 302, 338, 838, 724, 971, 725, 837, 864, 867, 868, 938, 941, 978, 980, 981	Li et al., 2014
Elbow helix-2	Detected by MOE within 5 Å of co-crystalized QZ-Val. These are: 692, 693, 694, 697, 824, 825, 828, 829, 832, 833, 987, 990, 991, 993, 994	Szewczyk et al, 2015
ATP binding site 1	426,427,429-431,471,551,552,583	NCBI protein database entry NP_035206.2; site name:“ATP binding site [chemical binding]”
ATP binding site 2	1069,1070,1072-1074,1114,1196,1197,1228	

All residues are identified according to their location in the mouse Abcb1 protein.

REFERENCES

Ferreira RJ, Ferreira MJ, dos Santos DJ. Molecular docking characterizes substrate-binding sites and efflux modulation mechanisms within P-glycoprotein. J Chem Inf Model 2013;53:1747–60.

Li J, Jaimes KF, Aller SG. Refined structures of mouse P-glycoprotein. Protein Sci. 2014;23:34–46.

Szewczyk P, Tao H, McGrath AP, Villaluz M, Rees SD, Lee SC, Doshi R, Urbatsch IL, Zhang Q, Chang G. Snapshots of ligand entry, malleable binding and induced helical movement in P-glycoprotein. Acta Cryst 2015;71:732–41.
