# Supplementary material for: Substrate-specific effects of pirinixic acid derivatives on ABCB1-mediated drug transport
Source: Oncotarget. 2016 Feb 12;7(10):11664–76. doi: 10.18632/oncotarget.7345 (PMC4905501; doi:10.18632/oncotarget.7345)
Supplement: Supplementary file 2 [file oncotarget-07-11664-s002.docx]

**Suppl. Table 8**. Effects of LP117 (2µM) or the known ABCB1 inhibitor verapamil (5µM) on the sensitivity of ABCB1-expressing UKF-NB-3^r^DOX^20^, UKF-NB-3^r^PCL^10^, and UKF-NB-3^r^VCR^10^ cells to the cytotoxic ABCB1 substrates doxorubicin, paclitaxel, and vincristine. Cell viability and concentrations that reduce cell viability by 50% (IC_50_) were determined after 120h of incubation by MTT assay.

| **UKF-NB-3^r^DOX^20^** | |  | |  |
| --- | --- | --- | --- | --- |
|  |  | IC_50_ (ng/mL) | |  |
| drug | viability in the presence of LP117  alone  (% control) | ABCB1 substrate alone | + LP117 | fold change^1^ |
| doxorubicin | 93 ± 12 | 74.51 ± 18.05 | 106.17 ± 30.22 | 0.7 |
| paclitaxel | 93 ± 12 | 59.22 ± 12.69 | 19.96 ± 4.52 | 3.0 |
| vincristine | 93 ± 12 | 18.85 ± 3.84 | 3.13 ± 0.96 | 6.0 |
|  |  |  |  |  |
|  |  | IC_50_ (ng/mL) | |  |
| drug | viability in the presence of verapamil  alone  (% control) | ABCB1 substrate alone | + verapamil | fold change |
| doxorubicin | 78 ± 20 | 74.51 ± 18.05 | 5.99 ± 2.10 | 12.4 |
| paclitaxel | 78 ± 20 | 59.22 ± 12.69 | 1.96 ± 0.46 | 30.2 |
| vincristine | 78 ± 20 | 18.85 ± 3.84 | 0.67 ± 0.19 | 28.1 |
|  |  |  |  |  |
| **UKF-NB-3^r^PCL^10^** |  |  |  |  |
|  |  | IC_50_ (ng/mL) | |  |
| drug | viability in the presence of LP117  alone  (% control) | ABCB1 substrate alone | + LP117 | fold change |
| doxorubicin | 92 ± 11 | 66.23 ± 19.81 | 86.56 ± 21.63 | 0.8 |
| paclitaxel | 92 ± 11 | 81.65 ± 17.79 | 33.22 ± 7.18 | 2.5 |
| vincristine | 92 ± 11 | 20.36 ± 4.55 | 4.91 ± 1.03 | 4.1 |
|  |  |  | |  |
| drug | viability in the presence of verapamil  alone  (% control) | ABCB1 substrate alone | + LP117 | fold change |
| doxorubicin | 81 ± 8 | 66.23 ± 19.81 | 7.37 ± 1.61 | 9.0 |
| paclitaxel | 81 ± 8 | 81.65 ± 17.79 | 2.22 ± 0.49 | 36.8 |
| vincristine | 81 ± 8 | 20.36 ± 4.55 | 1.22 ± 0.27 | 16.7 |

| **UKF-NB-3^r^VCR^10^** | |  | |  |
| --- | --- | --- | --- | --- |
|  |  | IC_50_ (ng/mL) | |  |
| drug | viability in the presence of LP117  alone  (% control) | ABCB1 substrate alone | + LP117 | fold change^1^ |
| doxorubicin | 100 ± 12 | 41.33 ± 1.31 | 35.34 ± 14.14 | 1.2 |
| paclitaxel | 100 ± 12 | 54.48 ± 16.47 | 5.77 ± 1.86 | 9.4 |
| vincristine | 100 ± 12 | 77.08 ± 19.0 | 1.17 ± 0.32 | 65.9 |
|  |  |  |  |  |
|  |  | IC_50_ (ng/mL) | |  |
| drug | viability in the presence of verapamil  alone  (% control) | ABCB1 substrate alone | + verapamil | fold change |
| doxorubicin | 93 ± 7 | 41.33 ± 1.31 | 7.28 ± 2.42 | 5.7 |
| paclitaxel | 93 ± 7 | 54.48 ± 16.47 | 2.83 ± 0.59 | 19.3 |
| vincristine | 93 ± 7 | 77.08 ± 19.0 | 0.99 ± 0.15 | 77.9 |

^1^ fold change (IC_50_ ABCB1 substrate/ IC_50_ ABCB1 substrate in the presence of LP117 or verapamil, respectively)
