# Supplementary material for: Substrate-specific effects of pirinixic acid derivatives on ABCB1-mediated drug transport
Source: Oncotarget. 2016 Feb 12;7(10):11664–76. doi: 10.18632/oncotarget.7345 (PMC4905501; doi:10.18632/oncotarget.7345)
Supplement: Supplementary file 3 [file oncotarget-07-11664-s003.docx]

**Suppl. Table 12**. Structures of the investigated compounds.

| Number | Compound | Structure |
| --- | --- | --- |
| 1 | HZ18 |  |
| 2 | HZ20 |  |
| 3 | HZ25 |  |
| 4 | HZ27 |  |
| 5 | HZ28 |  |
| 6 | HZ34 |  |
| 7 | HZ37 |  |
| 8 | HZ42 |  |
| 9 | HZ47 |  |
| 10 | HZ49 |  |
| 11 | HZ51 |  |
| 12 | HZ52 |  |
| 13 | HZ53 |  |
| 14 | HZ55 |  |
| 15 | HZ56 |  |
| 16 | HZ59 |  |
| 17 | HZ61 |  |
| 18 | HZ64 |  |
| 19 | HZ65 |  |
| 20 | HZ74 |  |
| 21 | HZ75 |  |
| 22 | HZ76 |  |
| 23 | HZ82 |  |
| 24 | HZ97 |  |
| 25 | LP105 |  |
| 26 | LP117 |  |
| 27 | LP119 |  |
| 28 | LP120 |  |
| 29 | LP121 |  |
| 30 | LP123 |  |
| 31 | YS49 |  |
| 32 | YS71 |  |
| 33 | YS78 |  |
| 34 | YS80 |  |
| 35 | YS81 |  |
| 36 | YS82 |  |
| 37 | YS83 |  |
| 38 | YS85 |  |
| 39 | YS121 |  |
